# Deprescribing opioids post-surgery – whose responsibility is it?

**DOI:** 10.1177/20494637231154370

**Published:** 2023-02-16

**Authors:** Neetu Bansal

**Affiliations:** Division of Pharmacy and Optometry, 5292 The University of Manchester, Manchester, UK

As a surgical pharmacist on the wards, I recall numerous heart-sinking occasions reviewing the medication of patients admitted to the hospital for a different complaint but still taking opioids initially prescribed during their previous admission for surgery. The question arises repeatedly in discussions with colleagues – whose responsibility is it to deprescribe opioids commenced post-operatively?

Although opioids remain the mainstay of post-operative pain management following both minor and major surgical procedures,^1^ there is a lack of robust evidence-based literature to guide the optimal analgesic medicine use (type and duration) and effective deprescribing strategies post-discharge. Opioid prescriptions after surgery are a risk factor for subsequent opioid dependence, which may be as high as 6.5% in previously opioid naïve patients.^2^ Currently, in the UK, we lack knowledge of the true prevalence of long-term opioid use after surgery.

With more complex surgical procedures being undertaken as a day case or short stay and the introduction of enhanced recovery programmes resulting in a shorter post-operative length of hospital stay, patients are no longer being fully weaned off their analgesics by the time of hospital discharge. The responsibility for managing post-operative analgesia may change hands from the surgeon to primary care without exploring the reasons for ongoing opioid therapy. Furthermore, discharging patients home with opioids poses a risk of unused opioids at home, hence the risk of illicit diversion to friends and family who may describe pain.^3^

In 2021, the UK Faculty of Pain Medicine issued guidance around the best practice of opioids and surgery.^4^ This document states that all healthcare professionals involved in surgery and peri-operative care must collaborate to ensure robust opioid stewardship.^4^ Currently, there is a lack of national guidance on peri-operative opioid prescribing. Furthermore shared decision-making in healthcare has been around for a long time.^5^ Increasing evidence has shown involving patients in decisions around their care leads to positive outcomes, and it is imperative to ensure peri-operative plans are drawn up collaboratively with patients.^6^

A collaborative effort of all healthcare teams across the secondary and primary care interfaces is needed to promote opioid stewardship. The focus should be on effective written communication to GPs on opioid tapering plans and any perceived problems and ensuring patients are provided with written information in the form of leaflets/pamphlets^7^ The emphasis should also be on all healthcare teams reciting the same mantra with opioid use and ensuring appropriate review at every encounter with the patient.
